# Ethical Leadership and Knowledge Sharing: The Impacts of Prosocial Motivation and Two Facets of Conscientiousness

**DOI:** 10.3389/fpsyg.2020.581236

**Published:** 2020-11-09

**Authors:** Zhichen Xia, Fan Yang

**Affiliations:** ^1^ Normal College, Changshu Institute of Technology, Changshu, China; ^2^ School of Education, Soochow University, Suzhou, China

**Keywords:** knowledge sharing, ethical leadership, prosocial motivation, achievement-striving, dutifulness

## Abstract

This study adopts the social learning theory to explain how and when ethical leadership can predict knowledge sharing in the context of Chinese higher education. We collected two-wave data from 302 postgraduate students from 38 scientific research teams in Chinese universities. The results of this study show that ethical leadership has a direct and positive effect on knowledge sharing, and prosocial motivation fully mediates this relationship. Moreover, the boundary conditions for such effects have affirmed the positive effects of dutifulness and the adverse effects of achievement-striving on the relationship between prosocial motivation and knowledge sharing. The indirect effects of ethical leadership on knowledge sharing are stronger when dutifulness is high and achievement-striving is low. Several theoretical and practical implications are provided by this study. It suggests that the role of prosocial motivation, in tandem with the two facets of conscientiousness, deserves to be highlighted when studying knowledge sharing behavior in correlation with ethical leadership.

## Introduction

Knowledge sharing is renowned as a critical factor in raising employee, team, and organizational productivity and creativity (Collins and Smith, 2006; Wang and Noe, 2010). Knowledge sharing refers to “acts of making knowledge available to others within the organization” (Ipe, 2003, p. 32). At the team level, knowledge sharing can lead to better team performance (Faraj and Sproull, 2000; Lee et al., 2010), as well as disseminate innovative ideas, improve team processes, foster innovation capacity, and promote competitive advantages (Kalling, 2003; Yi, 2009). However, knowledge sharing also presents a moral dilemma for individual team members as knowledge is quite different from any other resource. Specifically, knowledge acquisition requires an immense amount of time and effort, whereas knowledge sharing makes knowledge lose original value or unique privilege once shared with others (Renzl, 2008; Park et al., 2017). Team members must decide between sharing knowledge for the welfare of other people or the collective and hiding knowledge for intensifying their competitive advantages. That is to say, knowledge sharing is an action that involves a high level of risk, sacrifice, and donation. In the context of higher education, knowledge sharing is also essential for knowledge innovation and the completion of research programs. Therefore, it is necessary to investigate the antecedents of knowledge sharing, which can promote the willingness of team members to participate in the activities of knowledge sharing, thus facilitating team innovation and productivity.

Several kinds of research have demonstrated the pivotal influence of team leadership on knowledge sharing. These researches highlighted that leadership had a direct effect on knowledge sharing (Kim and Yun, 2015). For example, Srivastava et al. (2006) found that empowering leadership was positively related to knowledge sharing. Lee et al. (2010) argued that the leadership role of knowledge builder enhanced knowledge sharing in teams. Also, researchers examined the effect of the team’s leadership on knowledge sharing from the perspective of morality (Bavik et al., 2017; Lu et al., 2019). The studies revealed that ethical leadership could promote knowledge sharing according to the social learning theory (Brown and Treviño, 2006; Lee et al., 2010). It argues that followers pay attention to ethical leaders’ behaviors, identify ethical leaders as attractive and credible role models, and come to emulate the modeled behaviors of their ethical leaders. Ethical leadership is characterized by fairness, honesty, openness, values, and trustworthiness (Brown and Treviño, 2006), which involves the function of a moral person or a moral manager that both can promote knowledge sharing among their followers.

Different from other organizational scenarios, within a knowledge-based environment, the scientific research team purely focuses on knowledge innovation or knowledge production. Postgraduate students normally face challenges in conducting academic research (Bandura, 2001), due to the high social interconnectivity within higher education (Tierney and Farmer, 2002); maximizing the willingness of team members to share knowledge can improve team innovation and productivity. In the context of higher education, ethical leadership forces supervisors to provide a moral model for postgraduate students and accentuate the substantial impact that they can have on postgraduate students. Ethical leadership will convince postgraduate students that they should be concerned for and respect others and may encourage postgraduate students to participate in prosocial behaviors. Literature has provided evidence on the positive relationship between ethical leadership and knowledge sharing in work situations. However, considering the differentiated focus of work and the role of the supervisor, the ethical leadership may influence knowledge sharing differently in the higher education context. Therefore, we propose:

H1: Ethical leadership will positively predict knowledge sharing.

However, the influence mechanisms of ethical leadership on knowledge sharing still need to be explored. Previous researches have demonstrated that ethical leadership of leaders can change followers’ moral cognition, concerns, or values (Zhu et al., 2011; Sosik et al., 2014), which in turn stimulate followers to engage in behaviors that comply with moral standards (Mayer et al., 2009; Steinbauer et al., 2014). Thus, we propose that the relationship between ethical leadership and knowledge sharing may be explained by the mediating mechanism of prosocial motivation. Prosocial motivation refers to an individual’s desire to benefit other people (Grant and Berg, 2011). Rather than being a stable trait-like construct, prosocial motivation is malleable, and can be seen as a state-like construct in the present study. For example, Grant and Berg (2011) found that an individual’s prosocial motivation can be enhanced by effective leader. The study of Sosik et al. (2014) demonstrated that leaders’ attitudes, values, and behaviors exert considerable influence on followers, resulting in their conversion to the mindset of the influencer. Prosocial motivation, in turn, leads followers to engage in knowledge sharing. Prosocial motivation encourages individual exert extra effort to help others rather than act in their own-interest (DeConinck, 2015). Prosocial motivation highlights the greater value on protecting and promoting the welfare and benefits of others (Eva et al., 2019). People with high level of prosocial motivation are less individualistic and are more likely to have a greater tendency to engage in knowledge sharing. In the context of higher education, Nejati and Shafaei (2018) found that the ethical behavior of mentors and supervisors can develop the moral values of students. In fact, the engagement in knowledge sharing is more precisely predicts by the knowledge sharer’s motivation, willingness, and attitude (Cabrera and Cabrera, 2002; Gagné, 2009). Thus, we speculate the following hypothesis:

H2: Prosocial motivation mediates the relationship between ethical leadership and knowledge sharing.

While the influence of ethical leadership on knowledge sharing is proposed to occur through the mediating process of prosocial motivation, it is expected that not all postgraduate students are equally likely to participate in knowledge sharing behaviors. Motivations and behaviors are, of course, closely related, but it is a mistake to conflate the two. The contingent effects of individual personality traits on the relationship between ethical leadership and knowledge sharing may result in motivations and behaviors that do not correspond with one another. Several studies have found that an individual’s personality may influence the implementation of the knowledge sharing process (Lee et al., 2015). We focus on the characteristics of conscientiousness because conscientiousness affects the moral dilemma of knowledge sharing (Chae et al., 2019). Specifically, conscientiousness comprises two separate and primary dimensions of dutifulness and achievement-striving, with each having varying influences on individual behaviors (Moon, 2001; Dudley et al., 2006; Chae et al., 2019). Dutifulness, representing the other-centered dimension, dutiful individuals are tend to engage in the activities that contribute to the other people, organizations, or collective (Moon et al., 2008). On the contrary, achievement-striving representing the self-centered dimension, achievement-striving individuals appear unwilling to take part in self-expansive behaviors if they believe that these actions would harm their interests, advantage, or position (Costa and McCrae, 1992; Schneider et al., 1996). To explore the above-stated assertions, individuals who are primarily driven by other orientations are more likely to engage in knowledge sharing because they make helping as their duty, genuinely care about the interests of others, and they tend to be less concerning for their personal gain or loss (Korsgaard et al., 1997; Moon et al., 2008). Conversely, individuals who are primarily driven by self-orientation may feel reluctant to share knowledge to safeguard their competitiveness and interests (Marinova et al., 2013), they view their co-workers as potential competitors and believe that they can only be successful if their co-workers cannot attain their goals. Putting these expectations together, we expect that the two facets of conscientiousness might moderate the relation between prosocial motivation and knowledge sharing. Therefore, we propose:

H3(a): Dutifulness will moderate the relationship between prosocial motivation and knowledge sharing, such that prosocial motivation affects knowledge sharing more positively at higher levels rather than lower levels of dutifulness.
H3(b): Achievement-striving will moderate the relation between prosocial motivation and knowledge sharing, such that prosocial motivation affects knowledge sharing more positively with lower levels rather than higher levels of achievement-striving.

**Figure 1:**
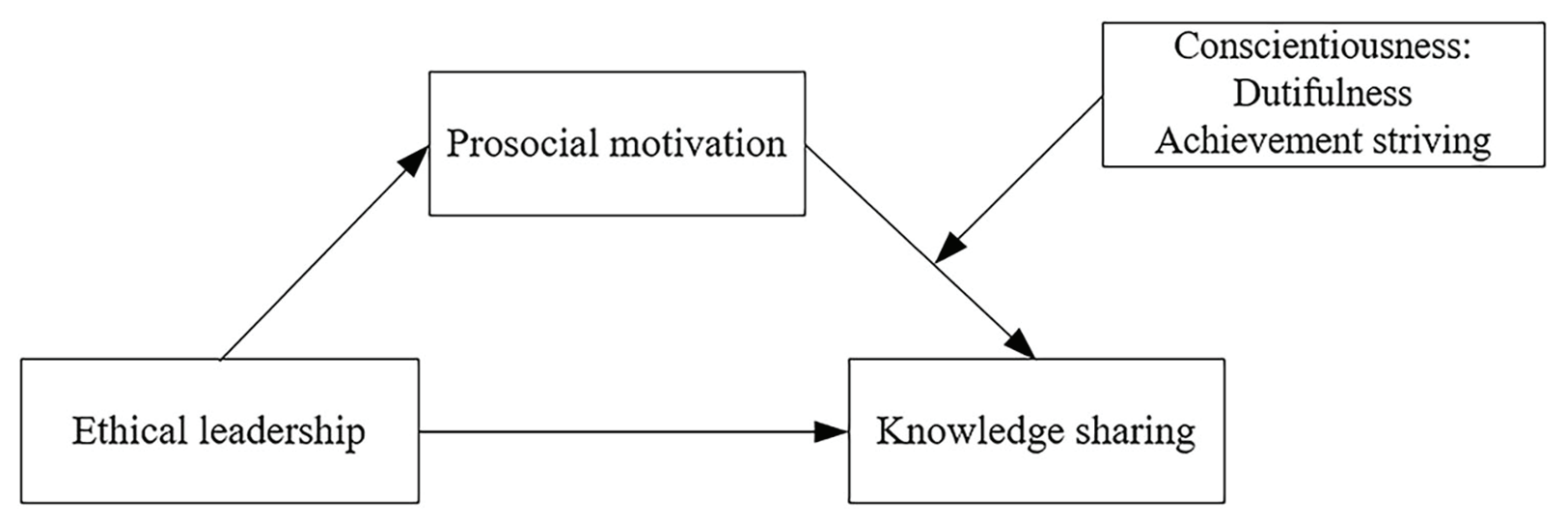
Hypothesized model.

To date, an examination of the moderating influence of the two facets of conscientiousness is still lacking, and previous studies have mainly regarded conscientiousness as a monolithic construct. Few studies considered the opposing implications of two distinct personality dimensions of conscientiousness (Chae et al., 2019). Therefore, the current study focuses on investigating the moderating effects of dutifulness and achievement-striving in relation to prosocial motivation and knowledge sharing. Ultimately, we developed a moderated-mediation model (Figure 1) whereby dutifulness and achievement-striving will conditionally influence the strength of the indirect effect between ethical leadership and knowledge sharing through prosocial motivation.

Above all, we put forward the following hypotheses:

H4(a): Dutifulness will moderate the indirect effect of ethical leadership on knowledge sharing *via* prosocial motivation, and the mediated relationship is stronger when dutifulness is higher.
H4(b): Achievement-striving will moderate the indirect effect of ethical leadership on knowledge sharing *via* prosocial motivation, and the mediated relationship is stronger when achievement-striving is lower.

In summary, previous studies have used ethical leadership, which is considered as the antecedent of knowledge sharing and reported its positive significant association with knowledge sharing in the workplace. For example, Tang et al. (2015) showed that ethical leadership has positive influences on knowledge sharing. A study by Bavik et al. (2017) demonstrated that ethical leadership matters in fostering knowledge sharing among full-time employees. Lu et al. (2019) found that ethical leadership can promote knowledge sharing among employees. Few studies attempt to relate ethical leadership to knowledge sharing in the context of higher education. Thus, we focus on the research within higher education to examine whether the relationship between ethical leadership and knowledge sharing is consistent with previous findings. Second, Bavik et al. (2017) realized that although they supported the mediating effects of controlled motivation in accounting for the relationship between ethical leadership and knowledge sharing, the mediating factor is not all-inclusive, which resulted in the call for verification of the roles of other types of motivation (e.g., introjected motivation and moral motivation) in the relationship between ethical leadership and knowledge sharing. In the present study, we identify prosocial motivation as the mediating mechanism between ethical leadership and knowledge sharing to echo their claim. Third, Lu et al. (2019) claim that, the effect of followers’ characteristics on the relationship between ethical leadership and knowledge sharing is still understudied. We explore the boundary conditions that may nullify the effectiveness of ethical leadership. Accordingly, our study aims to strengthen this relationship between ethical leadership and knowledge sharing and examine why and how ethical leadership predicts knowledge sharing in the context of higher education.

## Materials and Methods

### Sample and Procedure

Data were collected from postgraduate students from three universities in the city of Suzhou. Questionnaires were administered to 320 postgraduate students from 40 teams at two time points. Postgraduate students rated the ethical leadership of their supervisors, prosocial motivation, and conscientiousness at time 1. After a 4-week interval, supervisors assessed postgraduate students’ behaviors of knowledge sharing in their teams at time 2. The two data-source and data-wave was to avoid the common method bias (Podsakoff et al., 2003). In total, we obtained useable data from 302 team members and 38 teams after deleting missing and invalid questionnaires. All the response rates were over 94%. The average team size was 22.19 members per team; the average age of the team members was 24.61 years old (*SD* = 2.38), and 152 were male (50.50%). This study was approved by the Academic Ethical Group of The Faculty of Education, Soochow University, and the postgraduate students were informed of the processes and objectives driving the research.

### Measure

#### Ethical Leadership

Postgraduate students assessed the ethical leadership of their supervisors with six items adapted from ethical leadership scale of Cheng et al. (2004). Sample items are “My supervisor does not use guanxi (personal relationships) or back-door practices to obtain illicit personal gains.” “My supervisor employs people according to their virtues and does not envy others’ abilities and virtues.” “My supervisor does not take advantage of me for personal gain.” Six items range from 1 to 6, with 1 representing “not at all” and 6 representing “frequently.” Higher scores represent higher levels of ethical leadership. Liao et al. (2017) reported the Cronbach’s alpha coefficient for ethical leadership to be 0.826. We obtained a Cronbach’s alpha coefficient of 0.743 for the scale ratings in this study.

#### Knowledge Sharing

Supervisors rated postgraduate students’ knowledge sharing using four-item scale adapted from Lu et al. (2006). Sample items are “This postgraduate student shares with others useful work experience and know-how.” “This postgraduate student takes out his/her knowledge to share with more team members.” “This postgraduate student takes the initiative to share his/her work-related knowledge to his/her team members.” All four-item employed a seven-point Likert scale from “strongly disagree” to “strongly agree.” Higher scores indicate higher levels of knowledge sharing. Cronbach’s alpha coefficient was 0.768 in this study.

#### Prosocial Motivation

Postgraduate students rated prosocial motivation adapted from five-item scale of Grant and Sumanth (2009). Sample items are “I get energized by working on tasks that have the potential to benefit others.” “I do my best when I am working on a task that contributes to the well-being of others.” All five items were assessed on a seven-ponit scale, ranging from 1 (strongly disagree) to 7 (strongly agree). Higher scores represent higher levels of prosocial motivation. Cronbach’s alpha coefficient was 0.884 in this study.

#### Conscientiousness

We adopted the scales from five-item scale of Wang et al. (2011), the Chinese Big Five Personality Inventory Brief (CBF-PI-B) Version. We used three items for assessing dutifulness, and two items to evaluate achievement-striving. These two scales ranged from 1 (totally disagree) to 5 (totally agree). A sample item for dutifulness is “I try to perform all the tasks assigned to me conscientiously” and a sample item for achievement-striving is “I work hard to accomplish my goals.” We obtained Cronbach’s alpha coefficient of 0.761 and 0.737 for the two scales ratings used in this study.

#### Control Variables

We controlled for the effects of age, gender, majoring discipline, and team size. Demographic variables, such as age, gender, and major, were related to the assessment of knowledge sharing. Age was measured in years, gender (1 = female, 0 = male), and majoring disciplines [1 = science and engineering, 0 = others (e.g., social sciences, arts, and humanities)] were dummy coded. Team size was also controlled in the analysis because team size plays an essential role in interaction and dynamics among team members which may further influence knowledge sharing (Kwak and Jackson, 2015). Team size was indicated by the number of members in the team.

### Data Analysis

The theoretical framework was conceptualized at the individual level of analysis. However, the supervisors assessed the knowledge sharing behavior of approximately 6–10 postgraduate students (average number of postgraduate students per supervisor = 7.95). Given the nested data structure, we employed three criteria of ICC1, ICC2, and *r*_wg_ to validate the potential clustering effects of the dependent variable (knowledge sharing). Three values of ethical leadership were ICC1 = 0.03, ICC2 = 0.45, and *r*_wg_ = 0.53. According to standards of James (1982) and Schneider et al. (1998), when ICC1 < 0.12 and ICC2 < 0.47, the clustering effect was not significant, so the hypotheses of this study could be justified at the individual level.

We tested the hypotheses at the individual level with multiple linear regressions. In addition, the structural equation modeling approach was adopted to compare the model fit indices between the baseline model and a few alternative models. Then, we followed the suggestions proposed by Preacher et al. (2007) to examine the mediating effects of prosocial motivation using a bootstrap test. Moreover, we examined the moderated mediation according to procedures of Edwards and Lambert (2007).

## Results

### Preliminary Analyses

To examine the construct distinctiveness of the five variables (ethical leadership, prosocial motivation, dutifulness, achievement-striving, and knowledge sharing), we conducted a series of confirmatory factor analyses with Amos (see Table 1). Compared to other five models, the hypothesized five-factor model showed adequate fit indices (*χ* ^2^/*df* = 2.602, RMSEA = 0.073, NFI = 0.930, CFI = 0.991, and TLI = 0.952), which supported the construct distinctiveness of the variables, and the common method variance problem has been avoided.

**Table 1:** Model fit results for confirmatory factor analyses.

Model	*χ* ^2^	*df*	*χ* ^2^ */df*	RMSEA	NFI	CFI	TLI
Model 1: the hypothesized five-factor model	564.739	217	2.602	0.073	0.930	0.991	0.952
Model 2: four-factor model (dutifulness and achievement-striving are combined)	759.467	224	3.390	0.087	0.744	0.803	0.777
Model 3: three-factor model (ethical leadership, dutifulness, and achievement-striving are combined)	1026.050	227	4.520	0.108	0.654	0.706	0.672
Model 4: three-factor model (dutifulness, achievement-striving, and prosocial motivation are combined)	1020.504	227	4.496	0.108	0.656	0.708	0.674
Model 5: two-factor model (ethical leadership, dutifulness, achievement-striving, and prosocial motivation are combined)	1146.405	229	5.006	0.142	0.614	0.59	0.627
Model 6: single-factor model (all items are loaded on a single factor)	1222.834	230	5.317	0.149	0.588	0.634	0.598

As shown in Table 2, ethical leadership was significantly and positively related to knowledge sharing (*r* = 0.334, *p* < 0.01). Prosocial motivation was significantly and positively associated with ethical leadership (*r* = 0.441, *p* < 0.01) and knowledge sharing (*r* = 0.655, *p* < 0.01). Prosocial motivation was associated significantly and positively with dutifulness (*r* = 0.402, *p* < 0.01) and had no significant relation with achievement-striving (*r* = 0.054, *p* > 0.05). Knowledge sharing was associated significantly and positively with dutifulness (*r* = 0.366, *p* < 0.01) and had no significant relation with achievement-striving (*r* = 0.111, *p* > 0.05).

**Table 2:** Means, SD, and correlations among variables.

Variable		*M*	*SD*	1	2	3	4	5	6	7	8
1.	Gender^a^	0.505	0.501								
2.	Age	24.609	2.382	−0.035							
3.	Majoring discipline^b^	0.311	0.464	0.323^**^	0.074						
4.	Time size	22.195	25.725	−0.050	−0.087	−0.222^**^					
5.	Ethical leadership	4.456	0.663	−0.076	−0.005	0.019	−0.083				
6.	Prosocial motivation	5.757	0.826	0.012	0.028	0.006	−0.207^**^	0.441^**^			
7.	Dutifulness	3.848	0.568	−0.236^**^	−0.030	0.023	−0.217^**^	0.204^**^	0.402^**^		
8.	Achievement striving	3.515	0.479	−0.139^*^	0.044	0.058	−0.015	0.099	0.054	0.345^**^	
9.	Knowledge sharing	5.432	0.788	−0.194^**^	0.081	−0.012	−0.088	0.334^**^	0.655^**^	0.366^**^	0.111

** *p* < 0.01

* *p* < 0.05.

a 0 = male, 1 = female.

b 0 = science and engineering, 1 = others (social science, arts, and humanities).

### Main Effects and Mediation Effects

We used multiple linear regressions to test all hypotheses. The results of model 4 in Table 3 show that ethical leadership was significantly and positively related to knowledge sharing (*β* = 0.376, *p* < 0.001) after entering the control variables. Thus, hypothesis 1 was supported. In examining the mediation effect of prosocial motivation, the results of models 2 and 5 show that ethical leadership was positively related to prosocial motivation (*β* = 0.539, *p* < 0.001), prosocial motivation was positively related to knowledge sharing (*β* = 0.626, *p* < 0.001), and ethical leadership had no significant effect on knowledge sharing (*β* = 0.038, *p* > 0.05) after entering prosocial motivation. Besides, the bootstrapping test indicated that the indirect effect of ethical leadership on knowledge sharing *via* prosocial motivation was significant [*z* = 0.338; *SE* = 0.049; 95%CI (0.244, 0.439)]. These results showed that prosocial motivation fully mediated the positive effect of ethical leadership on knowledge sharing. Therefore, hypothesis 2 was supported.

**Table 3:** Results of regression analyses.

Variable	Prosocial motivation		Knowledge sharing			
	Model 1	Model 2	Model 3	Model 4	Model 5	Model 6
Gender	0.015	0.083	−0.343^***^	−0.296^**^	−0.348^***^	−0.294^***^
Age	0.006	0.009	0.023	0.025	0.019	0.023
Majoring discipline	−0.069	−0.095	0.073	0.055	0.114	0.111
Time size	−0.006^*^	−0.005^**^	−0.002	−0.001	0.002	0.001
Ethical leadership		0.539^***^		0.376^***^	0.038	0.026
Prosocial motivation					0.626^***^	0.618^***^
Dutifulness						0.085
Achievement striving						0.052
Prosocial motivation × Dutifulness						0.164^**^
Prosocial motivation × Achievement striving						−0.230^*^
*R* ^2^	0.041	0.477	0.054	0.153	0.486	0.508
*△R* ^2^	0.028	0.214	0.041	0.139	0.475	0.491
*F*	3.114^*^	17.217^***^	4.197^**^	10.600^***^	45.955^***^	29.762^***^

*n* = 302.

*** *p* < 0.001

** *p* < 0.01

* *p* < 0.05.

### Moderation by Two Facets of Conscientiousness

**Figure 2:**
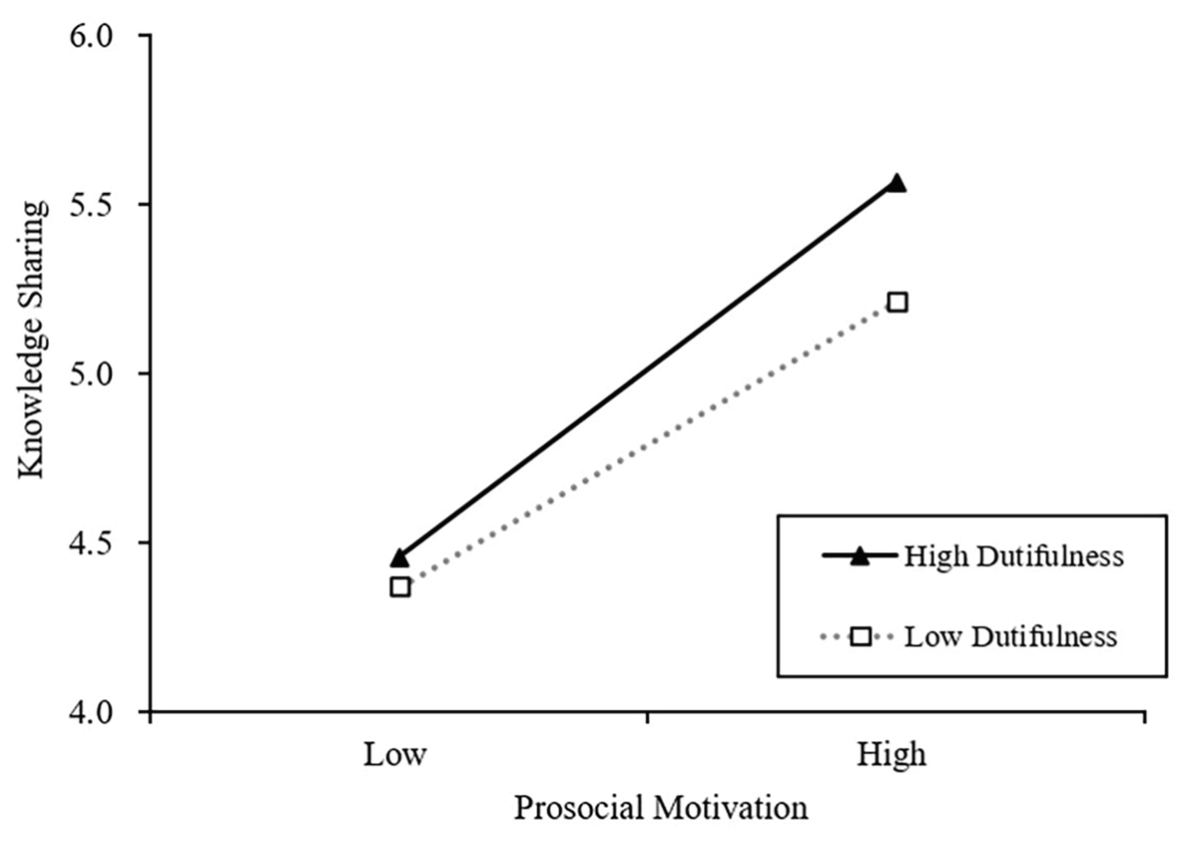
The moderating effect of dutifulness in the relation between prosocial motivation and knowledge sharing.

**Figure 3:**
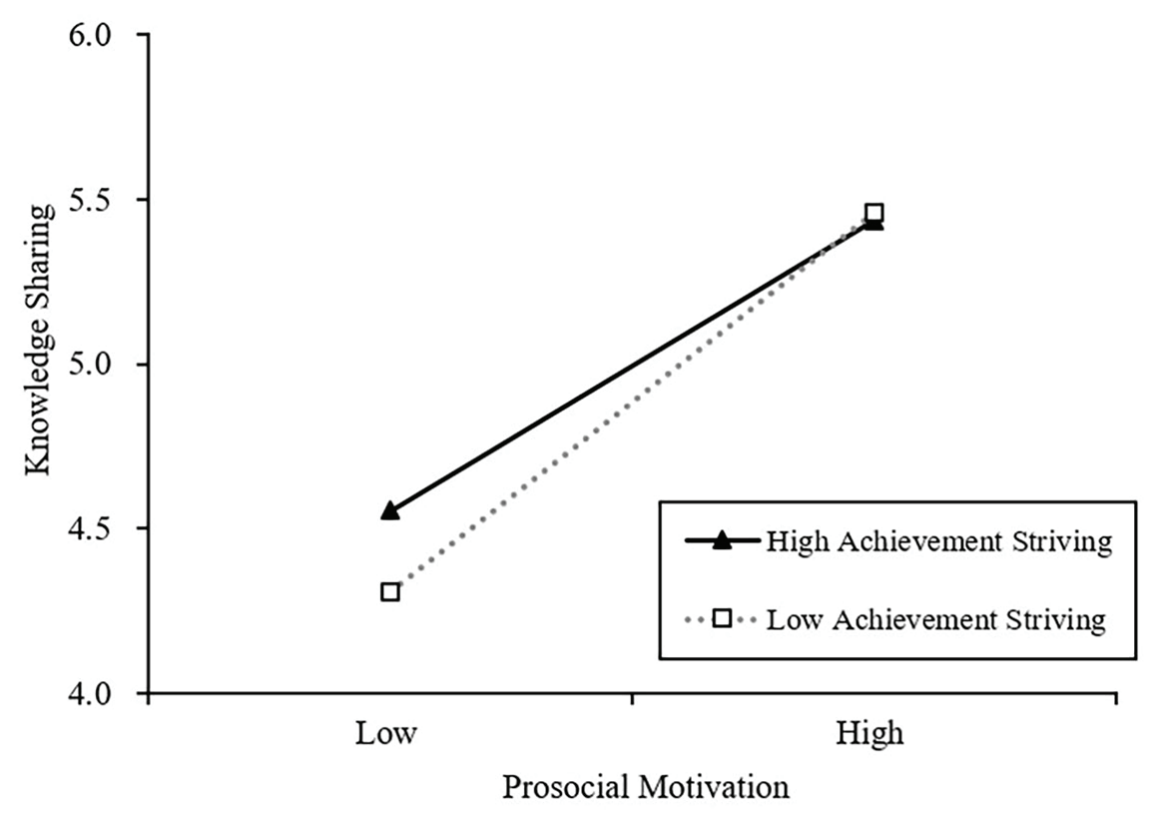
The moderating effect of achievement striving in the relation between prosocial motivation and knowledge sharing.

Hypothesis 3 proposes that dutifulness and achievement-striving have a moderating effect on the relation between prosocial motivation and knowledge sharing. As shown in model 6 in Table 3, the interaction term of “Prosocial motivation × Dutifulness” is positively related to knowledge sharing (*β* = 0.164, *p* < 0.01), while interaction term of “Prosocial motivation × Achievement-striving” is negatively associated with knowledge sharing (*β* = −0.230, *p* < 0.05). These results indicate that the two facets of conscientiousness moderate the effect of prosocial motivation against knowledge sharing. Following procedures of Aiken and West (1991), we conducted simple slope analyses to examine these interaction patterns. Figure 2 shows that prosocial motivation was positively related to knowledge sharing when dutifulness was high (one SD above the mean; *b* = 0.673, *p* < 0.001) but became relatively weaker when dutifulness was low (one SD below the mean; *b* = 0.512, *p* < 0.001). Meanwhile, Figure 3 shows that prosocial motivation was positively related to knowledge sharing when achievement-striving is low (one SD below the mean; *b* = 0.698, *p* < 0.001) but became relatively weaker when achievement-striving is high (one SD above the mean; *b* = 0.534, *p* < 0.001). Taken together, both hypotheses 3a and 3b were supported.

### Test of Moderated Mediation

Furthermore, hypothesis 4 assumes that dutifulness and achievement-striving moderate the mediation effect of prosocial motivation. We examined the moderated mediation, according to procedures of Edwards and Lambert (2007). The results revealed that the indirect effect was 0.297 with a 95%CI of (0.210, 0.394) when the dutifulness was low, vs. 0.364 with a 95%CI of (0.259, 0.503), when it was high. The difference between the two conditions was 0.067, with a 95%CI of (0.236, 0.428). The indirect effect was 0.375 with a 95%CI of (0.272, 0.498) when the achievement-striving was low, vs. 0.290 with a 95%CI of (0.188, 0.417), when it was high. The difference between the two conditions was −0.085, with a 95%CI of (0.203, 0.459). Therefore, hypotheses 4a and 4b were supported.

## Discussion

In the current research, we examine the relationship between ethical leadership and knowledge sharing, as well as the mediating role of prosocial motivation and the moderating role of the two facets of conscientiousness. In support of hypothesis 1, we found that ethical leadership had a direct positive prediction on knowledge sharing. According to social learning theory, followers identify an ethical role model, internalize the model’s attitude, and emulate modeled behavior by observing the ethical behaviors of their influencers (Bandura, 1986; Brown et al., 2005). Leader ethicality has demonstrated a positive relationship with the ethical behaviors exhibited by the followers (Newman et al., 2014; Wang and Sung, 2016). Moreover, knowledge sharing is generally represented as an ethical or voluntary act, which shares similarities with many other prosocial behaviors. Therefore, we revealed a positive relationship in the context of higher education that was also consistent with the previous researches.

In line with hypothesis 2, the results of the present research showed that prosocial motivation played a fully mediating role in the relationship between ethical leadership and knowledge sharing. As per the findings of the previous research, ethical leaders were typically identified as attractive and credible role models that can be related to prosocial attitudes or intentions of followers. Also, consistent with the theory of reasoned action (Fishbein and Ajzen, 1975), individuals’ motivation has been found to predict actual behavior. In other words, if an individual intends to do an act, then it is likely that the individual will do it. Thus, in this study, individuals’ prosocial motivation played a mediating role in the relationship between ethical leadership and knowledge sharing.

Additionally, our finding indicated that the two facets of conscientiousness moderated the relation between prosocial intention and knowledge sharing. As we proposed in hypothesis 3a, prosocial motivation affects knowledge sharing more positively with higher rather than lower levels of dutifulness. Further analysis also suggested that the indirect effects of ethical leadership on knowledge sharing are moderated by dutifulness. A highly dutiful individual possesses a strong sense of helping others (Moon et al., 2008). They are more likely to be prosocial and engage in behaviors that benefit others. In contrast, when an individual has a low level of dutifulness, the predicting effects of ethical leadership and prosocial motivation to share knowledge will be weakened. Thus, the knowledge sharing behaviors of postgraduate students increase with an increasing level of dutifulness in current study.

As we proposed in hypothesis 3b, prosocial motivation affects knowledge sharing more positively with lower rather than higher levels of achievement-striving. Moreover, further analysis also suggested that the indirect effects of ethical leadership on knowledge sharing are moderated by achievement-striving. Higher achievement-striving individuals focus more on self-interest and self-performance. They strive to win or outperform others and consider other people as potential competitors, which push them to reluctantly engage in behaviors that are risky and costly for their self-interest when it comes to knowledge sharing (Smither and Houston, 1992; Tangirala et al., 2013). Thus, the predicting effects of ethical leadership and prosocial motivation for postgraduate students to share knowledge will weaken in this context. However, when an individual has a low level of achievement-striving, they are more likely to engage in knowledge sharing because of the relatively weak, self-centered orientation. Accordingly, postgraduate students’ knowledge sharing behaviors decrease with increasing levels of achievement-striving.

### Theoretical Implications

Our research results provide considerable theoretical contributions to the knowledge sharing and higher education literature. First, this research supports the mediating role of prosocial motivation in the relationship between ethical leadership and knowledge sharing. The present study highlights the role of prosocial motivation in facilitating knowledge, which complements the empirical evidence of the antecedents of knowledge sharing. Second, although research has established that ethical leadership can influence knowledge sharing (Bavik et al., 2017; Lu et al., 2019), few studies on ethical leadership and knowledge sharing have considered the contingent effect of the individual personality traits. The present study contribution lies in the idea that the effect of ethical leadership on knowledge sharing is not evenly distributed across individuals when dutifulness and achievement-striving are considered. That indicates that knowledge sharing behaviors increase with increasing levels of dutifulness, and knowledge sharing behaviors decrease with an increasing level of achievement-striving. Third, numerous findings in various organizational settings have demonstrated that ethical leadership can effectively promote knowledge sharing. Our research enriches the findings of ethical leadership and knowledge sharing in the context of higher education, which promotes the research value of ethical leadership and knowledge sharing in scientific research organizations.

### Practical Implications

Our findings also offer several useful practical suggestions. First, as ethical leadership is one of the most prominent factors in predicting knowledge sharing; ethical leaders can promote knowledge sharing through role modeling of desirable traits or ethical behavior (Brown and Treviño, 2006). Thus, ethical leaders should serve as role models and make their followers identify, internalize, and emulate modeled behaviors. When recruiting, selecting, or promoting supervisors, the companies and organizations should focus on their moral identity. Moreover, training and incentive mechanisms can be applied to develop the ethical leadership of supervisors, for example, measures of morality should be included into supervisors’ performance appraisal (Zhou et al., 2015).

Second, except the influence of ethical leadership on knowledge sharing, our findings also suggest that prosocial motivation is critical to knowledge sharing. In the context of high education, supervisors need to cultivate the willingness of postgraduate students to share knowledge. Afterward, postgraduate students will be more willing to share knowledge even at the cost of self-interest. This also helps us understand the mechanism of knowledge sharing behaviors of socially oriented employees in the work context. Organizations should encourage the prosocial tendency of employees, reinforce the importance of moral principles, and enhance the employees’ intrinsic motivation to take others’ interests into consideration.

Third, given the moderating role of dutifulness and achievement-striving, individuals with higher levels of dutifulness are more likely to share knowledge. Whereas, individuals with higher levels of achievement-striving are not likely to share knowledge. Thus, team leaders should consider and evaluate the two facets of conscientiousness among postgraduate students: provide high dutiful postgraduate students with enough supervisor support and coworker support, supply achievement-striving postgraduate students with the opportunities and resources to show themselves (Chae et al., 2019). Moreover, team leaders should make sure that supervision and punishment are implemented to avoid knowledge hiding behaviors.

Fourth, our study can provide valuable insights into how to cope with problems opposite to knowledge sharing, such as knowledge hiding (Abdullah et al., 2019). First, supervisors should show more altruistic behaviors or provide ethical conduct examples that postgraduate students can imitate, and regulate their knowledge hiding behaviors in their daily behaviors. Second, supervisors may recruit those with other-oriented characteristic to inhibit knowledge hiding. Third, organizations might avoid knowledge hiding by regulating the influencing factors, including monetary rewards, personal development, and personal career growth (Lee et al., 2015). The results of our study are also meaningful for further enhancing the positive effects of knowledge sharing and reducing the negative effects of unethical leadership, such as relieving employees’ perception of work stress (Zhou et al., 2015), promoting group creativity, and improving financial performance (Bartol et al., 2009).

Fifth, universities’ scientific research teams belong to a kind of organizational form whose main content is knowledge innovation and knowledge production. In the era of knowledge economy, the research findings based on scientific research teams of universities are also applicable to companies and organizations (Bartol et al., 2009). We believe that leaders, whether they are in universities or companies, should abide by moral principles and treat their employees or postgraduate students with full respect. In hiring employees or postgraduate students, individual morality, personality, and thinking should be given attention, inhibiting recruitment based on competence alone. Moreover, relevant projects or programs should be designed to improve the ethical leadership of team leaders, thus, employees might be persuaded to imitate team leaders’ prosocial behaviors. This positive organizational climate could eventually promote cooperation and sharing behaviors among employees within the organization.

### Limitations and Future Directions

Our study has several limitations that should be considered and addressed in the future. First, to reduce common method bias and advance our research, we measured postgraduate student ratings of knowledge sharing’s antecedent, mediator, moderate, and utilized supervisor ratings of knowledge sharing at two different time points. However, our research design makes it impossible to speculate about the causal relationships between variables. Thus, future studies should collect data apart from self-report measures, such as by using an experimental design to validate the causal effect of ethical leadership on knowledge sharing, the mediating effect of prosocial motivation, and the moderating effect of the two facets of conscientiousness.

Second, our findings suggested that prosocial motivation was a full mediator, indicating that the factors of individual cognition can be a driving force in facilitating an individual’s knowledge sharing behavior. However, we are not clear whether other mediating factors might play a similar role in strengthening knowledge sharing or diminish knowledge hiding. Several factors, such as individual moral identify, self-control, instrumental thinking, employees’ quality of relationship with organizational members, psychological safety, psychological empowerment, and organizational concern, have proved to act as mediators in the relationship between ethical leadership and knowledge sharing/hiding (Moran, 2005; Bavik et al., 2017; Men et al., 2018; Lu et al., 2019). Future studies should explore relevant variables that may mediate the relationships studied herein and the other practical impact of knowledge sharing/hiding.

Third, we only chose the knowledge sharing of postgraduate students as the dependent variable to measure the effects of ethical leadership. However, given that the ethical leadership of supervisors was closely related with supervisors’ knowledge sharing, and supervisors and postgraduate students may have different views and ideas on knowledge sharing. Based on this, future research should extend the present by incorporating different types of knowledge sharing, such as supervisors’ and postgraduate students’ knowledge sharing into the effects of ethical leadership, and delve into the mechanisms of ethical leadership on those knowledge sharing behaviors.

Finally, the questionnaires were distributed in Chinese universities, and the team members were postgraduate students in universities in one province in China, the sample size was moderate, which may weaken the generalizability of the results. Influenced by the traditional Confucian culture, Chinese people usually pay more attention to the spirit of collectivism and dedication, which may affect the accuracy of conscientious measurement. In this regard, future research should further replicate and examine the effects of ethical leadership on knowledge sharing and its influence mechanism in other organization, institutional, countries, or cultural contexts.

## Data Availability Statement

All datasets presented in this study are included in the article/Supplementary Material.

## Ethics Statement

The studies involving human participants were reviewed and approved by Academic Ethical Group of The Faculty of Education, Soochow University. The participants provided their written informed consent to participate in this study.

## Author Contributions

Both the authors listed have made a substantial, direct and intellectual contribution to the work, and approved it for publication.

### Conflict of Interest

The authors declare that the research was conducted in the absence of any commercial or financial relationships that could be construed as a potential conflict of interest.

## References

[ref1] Abdullah M. I. Dechun H. Ali M. Usman M. (2019). Ethical leadership and knowledge hiding: a moderated mediation model of relational social capital, and instrumental thinking. Front. Psychol. 10:2403. 10.3389/fpsyg.2019.02403, PMID: 31708841 PMC6823209

[ref2] Aiken L. S. West S. G. (1991). Multiple regression: Testing and interpreting interactions. Thousand Oaks, CA: Sage Publications, Inc.

[ref3] Bandura A. (1986). Social foundations of thought and action. Englewood Cliffs, NJ: Prentice-Hall.

[ref4] Bandura A. (2001). Social cognitive theory: an agentic perspective. Annu. Rev. Psychol. 52, 1–26. 10.1146/annurev.psych.52.1.1, PMID: 11148297

[ref5] Bartol K. M. Liu W. Zeng X. Wu K. (2009). Social exchange and knowledge sharing among knowledge workers: the moderating role of perceived job security. Manag. Organ. Rev. 5, 223–240. 10.1111/j.1740-8784.2009.00146.x

[ref6] Bavik Y. L. Tang P. M. Shao R. Lam L. W. (2017). Ethical leadership and employee knowledge sharing: exploring dual-mediation paths. Leadersh. Q. 29, 322–332. 10.1016/j.leaqua.2017.05.006

[ref7] Brown M. E. Treviño L. K. (2006). Ethical leadership: a review and future directions. Leadersh. Q. 17, 595–616. 10.1016/j.leaqua.2006.10.004

[ref8] Brown M. E. Treviño L. K. Harrison D. (2005). Ethical leadership: a social learning perspective for construct development and testing. Organ. Behav. Hum. Decis. Process. 97, 117–134. 10.1016/j.obhdp.2005.03.002

[ref9] Cabrera A. Cabrera E. F. (2002). Knowledge-sharing dilemmas. Organ. Stud. 23, 687–710. 10.1177/0170840602235001

[ref10] Chae H. Park J. Choi J. N. (2019). Two facets of conscientiousness and the knowledge sharing dilemmas in the workplace: contrasting moderating functions of supervisor support and coworker support. J. Organ. Behav. 40, 387–399. 10.1002/job.2337

[ref11] Cheng B. S. Chou L. F. Wu T. Y. Huang M. P. Farh J. L. (2004). Paternalistic leadership and subordinate responses: establishing a leadership model in Chinese organizations. Asian J. Psychol. 7, 89–117. 10.1111/j.1467-839X.2004.00137.x

[ref12] Collins C. Smith K. G. (2006). Knowledge exchange and combination: the role of human resource practices in the performance of high-technology firms. Acad. Manag. J. 49, 544–560. 10.5465/amj.2006.21794671

[ref13] Costa P. T. Jr. McCrae R. R. (1992). Revised NEO personality inventory (NEO-PI-R) and NEO five-factor inventory (NEO-FFI) professional manual. Odessa: Psychological Assessment Resources.

[ref14] DeConinck J. B. (2015). Outcomes of ethical leadership among salespeople. J. Bus. Res. 68, 1086–1093. 10.1016/j.jbusres.2014.10.011

[ref15] Dudley N. M. Orvis K. A. Lebiecki J. E. Cortina J. M. (2006). A meta analytic investigation of conscientiousness in the prediction of job performance: examining the intercorrelations and the incremental validity of narrow traits. J. Appl. Psychol. 91, 40–57. 10.1037/0021-9010.91.1.40, PMID: 16435937

[ref16] Edwards J. R. Lambert L. S. (2007). Methods for integrating moderation and mediation: a general analytical framework using moderated path analysis. Psychol. Methods 12, 1–22. 10.1037/1082-989X.12.1.1, PMID: 17402809

[ref17] Eva N. Newman A. Zhou A. J. Zhou S. S. (2019). The relationship between ethical leadership and employees’ internal and external community citizenship behaviors: the mediating role of prosocial motivation. Pers. Rev. 10.1108/PR-01-2019-0019 [Epub ahead of print]

[ref18] Faraj S. Sproull L. (2000). Coordinating expertise in software development teams. Manag. Sci. 46, 1554–1568. 10.1287/mnsc.46.12.1554.12072

[ref19] Fishbein M. Ajzen I. (1975). Belief, attitude, intention, and behavior: An introduction to theory and research. Philippines: Addison-Wesley Publishing Company.

[ref20] Gagné M. (2009). A model of knowledge-sharing motivation. Hum. Resour. Manag. 48, 571–589. 10.1002/hrm.20298

[ref21] Grant A. Berg J. (2011). “Prosocial motivation at work: when, why, and how making a difference makes a difference” in Oxford handbook of positive organizational scholarship. eds. Cameron K. S. Spreitzer G. M. (New York, NY: Oxford University Press), 28–44.

[ref22] Grant A. M. Sumanth J. J. (2009). Mission possible? The performance of prosocially motivated employees depends on manager trustworthiness. J. Appl. Psychol. 94, 927–944. 10.1037/a0014391, PMID: 19594235

[ref23] Ipe M. (2003). Knowledge sharing in organizations: a conceptual framework. Hum. Resour. Dev. Rev. 2, 337–359. 10.1177/1534484303257985

[ref24] James L. R. (1982). Aggregation bias in estimates of perceptual agreement. J. Appl. Psychol. 67, 219–229. 10.1037/0021-9010.67.2.219

[ref25] Kalling T. (2003). Knowledge management and the occasional links with performance. J. Knowl. Manag. 7, 67–81. 10.1108/13673270310485631

[ref26] Kim S. L. Yun S. (2015). The effect of coworker knowledge sharing on performance and its boundary conditions: an interactional perspective. J. Appl. Psychol. 100, 575–582. 10.1037/a0037834, PMID: 25198095

[ref27] Korsgaard M. A. Meglino B. M. Lester S. W. (1997). Beyond helping: do other-oriented values have broader implications in organizations? J. Appl. Psychol. 82, 160–177. 10.1037/0021-9010.82.1.160

[ref28] Kwak W. J. Jackson C. L. (2015). Relationship building in empowering leadership processes: a test of mediation and moderation. J. Manag. Organ. 21, 369–387. 10.1017/jmo.2015.11

[ref29] Lee P. Gillespie N. Mann L. Wearing A. (2010). Leadership and trust: their effect on knowledge sharing and team performance. Manag. Learn. 41, 473–491. 10.1177/1350507610362036

[ref30] Lee S. Yoo Y. Yun S. (2015). Sharing my knowledge? An interactional perspective. J. Manag. Psychol. 8, 986–1002. 10.1108/JMP-11-2013-0355

[ref31] Liao S. H. Widowati R. Hu D. C. Tasman L. (2017). The mediating effect of psychological contract in the relationships between paternalistic leadership and turnover intention for foreign workers in Taiwan. Asia Pac. Manag. Rev. 22, 80–87. 10.1016/j.apmrv.2016.08.003

[ref32] Lu L. Leung K. Koch P. T. (2006). Managerial knowledge sharing: the role of individual, interpersonal, and organizational factors. Manag. Organ. Rev. 2, 15–41. 10.1111/j.1740-8784.2006.00029.x

[ref33] Lu X. Zhou H. Chen S. (2019). Facilitate knowledge sharing by leading ethically: the role of organizational concern and impression management climate. J. Bus. Psychol. 34, 539–553. 10.1007/s10869-018-9555-8

[ref34] Marinova S. V. Moon H. Kamdar D. (2013). Getting ahead or getting along? The two-facet conceptualization of conscientiousness and leadership emergence. Organ. Sci. 24, 1257–1276. 10.1287/orsc.1120.0781

[ref35] Mayer D. M. Kuenzi M. Greenbaum R. Bardes M. Salvador R. B. (2009). How low does ethical leadership flow? Test of a trickle-down model. Organ. Behav. Hum. Decis. Process. 108, 1–13. 10.1016/j.obhdp.2008.04.002

[ref36] Men C. Fong P. S. W. Huo W. Zhong J. Jia R. Luo J. (2018). Ethical leadership and knowledge hiding: a moderated mediation model of psychological safety and mastery climate. J. Bus. Ethics 10.1007/s10551-018-4027-7 [Epub ahead of print]

[ref37] Moon H. (2001). The two faces of conscientiousness: duty and achievement striving in escalation of commitment dilemmas. J. Appl. Psychol. 86, 533–540. 10.1037/0021-9010.86.3.535, PMID: 11419812

[ref38] Moon H. Kamdar D. Mayer D. M. Takeuchi R. (2008). Me or we? The role of personality and justice as other-centered antecedents to innovative citizenship behaviors within organizations. J. Appl. Psychol. 93, 84–94. 10.1037/0021-9010.93.1.84, PMID: 18211137

[ref39] Moran P. (2005). Structural vs. relational embeddedness: social capital and managerial performance. Strateg. Manag. J. 26, 1129–1151. 10.1002/smj.486

[ref40] Nejati M. Shafaei A. (2018). Leading by example: the influence of ethical supervision on students’ prosocial behavior. High. Educ 75, 75–89. 10.1007/s10734-017-0130-4

[ref41] Newman A. Kiazad K. Miao Q. Cooper B. (2014). Examining the cognitive and affective trust-based mechanisms underlying the relationship between ethical leadership and organisational citizenship: a case of the head leading the heart? J. Bus. Ethics 123, 113–123. 10.1007/s10551-013-1803-2

[ref42] Park J. Chae H. Choi J. (2017). The need for status as a hidden motive of knowledge-sharing behavior: an application of costly signaling theory. Hum. Perform. 30, 21–37. 10.1080/08959285.2016.1263636

[ref43] Podsakoff P. M. MacKenzie S. B. Lee J. Y. Podsakoff N. P. (2003). Common method biases in behavioral research: a critical review of the literature and recommended remedies. J. Appl. Psychol. 85, 879–903. 10.1037/0021-9010.88.5.879, PMID: 14516251

[ref44] Preacher K. J. Rucker D. D. Hayes A. F. (2007). Addressing moderated mediation hypotheses: theory, methods, and prescriptions. Multivar. Behav. Res. 42, 185–227. 10.1080/00273170701341316, PMID: 26821081

[ref45] Renzl B. (2008). Trust in management and knowledge sharing: the mediating effects of fear and knowledge documentation. Omega 36, 206–220. 10.1016/j.omega.2006.06.005

[ref46] Schneider R. J. Hough L. M. Dunnette M. D. (1996). Broadsided by broad traits: how to sink science in five dimensions or less. J. Organ. Behav. 17, 639–655. 10.1002/(SICI)1099-1379(199611)17:6<639::AID-JOB3828>3.0.CO;2-9

[ref47] Schneider B. White S. S. Paul M. C. (1998). Linking service climate and customer perceptions of service quality: tests of a causal model. J. Appl. Psychol. 83, 150–163. 10.1037/0021-9010.83.2.150, PMID: 9577232

[ref48] Smither R. D. Houston J. M. (1992). The nature of competitiveness: the development and validation of the competitiveness index. Educ. Psychol. Meas. 52, 407–418. 10.1177/0013164492052002016

[ref49] Sosik J. J. Chun J. U. Zhu W. (2014). Hang on to your ego: the moderating role of leader narcissism on relationships between leader charisma and follower psychological empowerment and moral identity. J. Bus. Ethics 120, 65–80. 10.1007/s10551-013-1651-0

[ref50] Srivastava A. Bartol K. M. Locke E. A. (2006). Empowering leadership in management teams: effects on knowledge sharing, efficacy, and team performance. Acad. Manag. J. 49, 1239–1251. 10.5465/amj.2006.23478718

[ref51] Steinbauer R. Renn R. W. Taylor R. R. Njoroge P. K. (2014). Ethical leadership and followers’ moral judgment: the role of followers’ perceived accountability and self-leadership. J. Bus. Ethics 120, 381–392. 10.1007/s10551-013-1662-x

[ref52] Tang P. M. Bavik Y. L. Chen Y. F. Tjosvold D. (2015). “Linking ethical leadership to knowledge sharing and knowledge hiding: the mediating role of psychological engagement” in *International Proceedings of Economics Development and Research*; March 25, 2015; *Vol. 84.* 71–76.

[ref53] Tangirala S. Kamdar D. Venkataramani V. Parke M. R. (2013). Doing right versus getting ahead: the effects of duty and achievement orientations on employees’ voice. J. Appl. Psychol. 98, 1040–1050. 10.1037/a0033855, PMID: 23915430

[ref54] Tierney P. Farmer S. M. (2002). Creative self-efficacy: its potential antecedents and relationship to creative performance. Acad. Manag. J. 45, 1137–1148. 10.2307/3069429

[ref55] Wang M. C. Dai X. Y. Yao S. Q. (2011). Development of the Chinese big five personality inventory (CBF-PI) III: psychometric properties of CBF-PI brief version. Chin. J. Clin. Psychol. 19, 454–457. 10.16128/j.cnki.1005-3611.2011.04.004

[ref56] Wang S. Noe R. A. (2010). Knowledge sharing: a review and directions for future research. Hum. Resour. Manag. Rev. 20, 115–131. 10.1016/j.hrmr.2009.10.001

[ref57] Wang Y. D. Sung W. C. (2016). Predictors of organizational citizenship behavior: ethical leadership and workplace jealousy. J. Bus. Ethics 135, 117–128. 10.1007/s10551-014-2480-5

[ref58] Yi J. (2009). A measure of knowledge sharing behavior: scale development and validation. Knowl. Manag. Res. Pract. 7, 65–81. 10.1057/kmrp.2008.36

[ref60] Zhou H. Jin M. Ma Q. (2015). Remedy for work stress: the impact and mechanism of ethical leadership. Cent. Eur. J. Public Health 23, 176–180. 10.21101/cejph.a4246, PMID: 26851431

[ref61] Zhu W. Riggio R. E. Avolio B. J. Sosik J. J. (2011). The effect of leadership on follower moral identity: does transformational/transactional style make a difference? J. Leadersh. Org. Stud. 18, 150–163. 10.1177/1548051810396714

